# Henle fiber layer thickening and deficits in objective retinal function in participants with a history of multiple traumatic brain injuries

**DOI:** 10.3389/fneur.2024.1330440

**Published:** 2024-02-06

**Authors:** Elizabeth A. Stern-Green, Kelly R. Klimo, Elizabeth Day, Erica R. Shelton, Matthew L. Robich, Lisa A. Jordan, Julie Racine, Dean A. VanNasdale, Catherine E. McDaniel, Phillip T. Yuhas

**Affiliations:** ^1^College of Optometry, The Ohio State University, Columbus, OH, United States; ^2^Department of Ophthalmology, Nationwide Children’s Hospital, Columbus, OH, United States

**Keywords:** traumatic brain injury, electroretinography, optical coherence tomography, Henle fiber layer, scanning laser polarimetry, macula

## Abstract

**Introduction:**

This study tested whether multiple traumatic brain injuries (TBIs) alter the structure of the Henle fiber layer (HFL) and degrade cell-specific function in the retinas of human participants.

**Methods:**

A cohort of case participants with multiple TBIs and a cohort of pair-matched control participants were prospectively recruited. Directional optical coherence tomography and scanning laser polarimetry measured HFL thickness and phase retardation, respectively. Full-field flash electroretinography (fERG) assessed retinal function under light-adapted (LA) 3.0, LA 30 Hz, dark-adapted (DA) 0.01, DA 3.0, and DA 10 conditions. Retinal imaging and fERG outcomes were averaged between both eyes, and paired t-tests or Wilcoxon signed-rank tests analyzed inter-cohort differences.

**Results:**

Global HFL thickness was significantly (*p* = 0.02) greater in cases (8.4 ± 0.9 pixels) than in controls (7.7 ± 1.1 pixels). There was no statistically significant difference (*p* = 0.91) between the cohorts for global HFL phase retardation. For fERG, LA 3.0 a-wave amplitude was significantly reduced (*p* = 0.02) in cases (23.5 ± 4.2 μV) compared to controls (29.0 ± 8.0 μV). There were no other statistically significant fERG outcomes between the cohorts.

**Discussion:**

In summary, the HFL thickens after multiple TBIs, but phase retardation remains unaltered in the macula. Multiple TBIs may also impair retinal function, indicated by a reduction in a-wave amplitude. These results support the potential of the retina as a site to detect TBI-associated pathology.

## 1 Introduction

A traumatic brain injury (TBI) is an alteration in brain structure or function caused by an external force (1). TBI is prevalent worldwide, affecting approximately 55 million people (2). Having a prior TBI is a predictor for having another (3), and 35% of TBIs in athletes are repeat injuries (4). Repeated TBIs are a risk factor for developing chronic traumatic encephalopathy, a progressive neurodegeneration that compromises neuronal axons and that causes a syndrome of mood disorders with behavioral and cognitive impairments (5).

As an extension of the central nervous system, the retina has the potential to be a site for the detection of pathology associated with multiple TBIs. Most investigations on the retinal manifestations of TBI have assessed retinal ganglion cells (RGCs). The long axons of RGCs are likely subject to the shearing forces associated with TBI (6), and primary neuronal damage in the brain may be able to spread to RGCs through trans-synaptic degeneration (7). Clinical studies that report retinal nerve fiber layer thinning in subjects with a history of multiple TBIs provide evidence of RGC susceptibility (8, 9). RGCs are not the only retinal neurons with long axons that may be vulnerable to TBI pathology, however. Photoreceptors in the central macula send axons through the Henle fiber layer (HFL) to the outer plexiform layer (10). Directional optical coherence tomography (OCT), where the imaging beam is offset from the center of the pupil, can quantify the thickness of the HFL (11). Furthermore, the HFL is a birefringent, tissue because microtubules contained within photoreceptor axons change the index of refraction of the HFL based on the polarization status and on the propagation direction of light passing through it (12). Imaging techniques, like scanning laser polarimetry (SLP), that assess the phase retardation of polarized light, which is caused by the brifriengent properties of microtubules in the HFL, can parse the presence of photoreceptor axons from supporting Müller cells (13). It is unknown whether TBIs alter the thickness or the phase retardation of the HFL.

The objective function of the human retina after multiple TBIs is also poorly characterized. Tzekov and colleagues used electroretinography (ERG) to demonstrate a reduced photopic negative response amplitude, a marker of RGC function (14), in a mouse model of repeated TBI (15), but this finding was not replicated in a human population with a history of multiple TBIs (16). Moreover, Freed and Hellerstein did not find abnormalities in the aspects of the full-field flash ERG (fERG) waveform driven by photoreceptors and bipolar cells in participants with a “documented mild TBI” (17). The objective function of photoreceptors and bipolar cells after multiple TBIs remains unelucidated.

These knowledge gaps currently limit the ability of clinicians and of researchers to detect and to monitor pathology associated with multiple TBIs in the retina. Thus, the purpose of the current study is to test whether multiple TBIs alter the structure of the HFL and degrade cell-specific function in the retinas of human subjects with a history of multiple TBIs.

## 2 Materials and methods

The work reported here is part of a larger project that sought to characterize retinal structure and function in participants with a history of multiple traumatic brain injuries. Some of the results of this larger study already have been reported (16).

### 2.1 Participant identification

Two cohorts were recruited from The Ohio State University (OSU) optometry clinics and from the university at-large. A case cohort contained participants with a history of multiple mild–moderate TBIs, classified according to the Veterans Affairs/Department of Defense Clinical Practice Guideline for Management of Concussion/Mild Traumatic Brain Injury (18). The OSU Traumatic Brain Injury Identification Method (OSU TBI-ID), a validated assessment of lifetime TBI history (19), quantified the number of and the timing of TBI events. Chart reviews cross-referenced the results of the OSU TBI-ID. Healthy control participants were pair-matched to case subjects based on age and sex. Both cohorts participated in the same study protocol.

Inclusion criteria for case participants included: age ≥ 18 years; tobacco non-user; at least two lifetime mild–moderate TBIs; no lifetime severe TBIs; no diabetes mellitus or neurological diseases, except TBI; corrected visual acuity of 20/30 or better in each eye; maximum intraocular pressure of ≤21 mmHg in each eye; and no anterior or posterior segment diseases. Inclusion criteria for control participants were the same, save that they could have no lifetime history of TBI. Slit lamp biomicroscopy, Goldmann applanation tonometry, and a dilated fundus examination confirmed adherence to these criteria.

### 2.2 Retinal imaging

Two sets of retinal images were collected from both eyes during a single study session. Pupils were dilated prior to imaging. Detailed descriptions of the directional OCT and SLP protocols are available elsewhere (13). The directional OCT protocol is validated (20) and repeatable (21).

#### 2.2.1 Directional OCT

##### 2.2.1.1 Device and image acquisition

**Figure 1:**
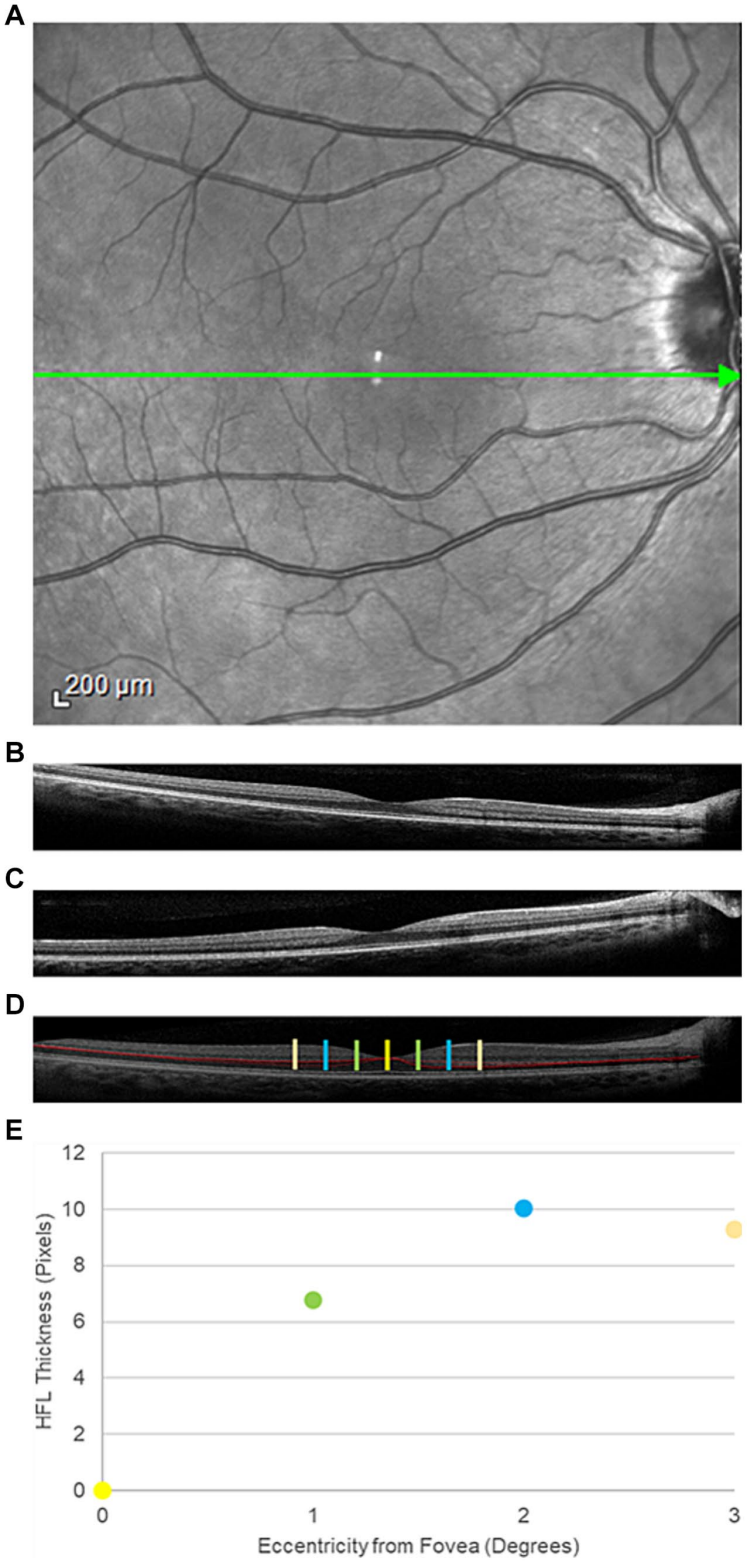
Representative directional optical coherence tomography images from a participant in the traumatic brain injury cohort. **(A)** *An face* image of the posterior pole. The green arrow through the center of the macula represents the orientation of interest. **(B)** Temporal and **(C)** nasal offset of the imaging beam through the pupil, resulting in visualization of the Henle fiber layer (HFL) to the contralateral side. **(D)** Composite image of the posterior pole with the HFL segmented in red. The yellow bar is the center of the fovea. The green bars mark 1° temporal (left) and nasal (right) from the fovea. The blue and orange bars mark positions 2° and 3° from the fovea, respectively. **(E)** HFL thickness profile, averaged across all four meridians (nasal, temporal, superior, and inferior) at the four positions within the central macula.

A single Heidelberg Spectralis spectral-domain OCT (Heidelberg Engineering, Heidelberg, Germany) generated 30° vertical and horizontal cross-sectional OCT images, centered on the fovea. Three cross-sectional images were acquired in both the horizontal and vertical orientations (Figure 1A). For the first image in each orientation, the imaging beam was aligned with the optical axis, creating an image where retinal displacement was symmetric around the fovea. For the two additional images, the imaging beam was offset by 3 mm on either side of the foveal-centered optical axis position, allowing for visualization of the HFL on the contralateral side of the offset (Figures 1B,C). A piece of grid paper placed at the base of the instrument helped guide the device to the correct offset position.

##### 2.2.1.2 Image processing

Images with a quality metric > 20 dB were retained for analysis. A single masked observer manually segmented the HFL in the cross-sectional offset OCT images using Adobe Photoshop (Adobe, San Jose, CA). Offset images in the same orientation, but opposite offset, were combined to create a single image, which contained the complete, segmented HFL (Figure 1D). Custom MATLAB programming (Mathworks, Natick, MA) quantified the thickness of the HFL in pixels in the temporal, superior, nasal, and inferior meridians. Meridional HFL values then were averaged into a single HFL thickness profile for each eye (Figure 1E). HFL thickness was measured globally and at 1° intervals from 1° to 3° eccentricity from the center of the fovea. Central foveal thickness also was measured using commercial software on the device.

#### 2.2.2 Scanning laser polarimetry

##### 2.2.2.1 Device and image acquisition

**Figure 2:**
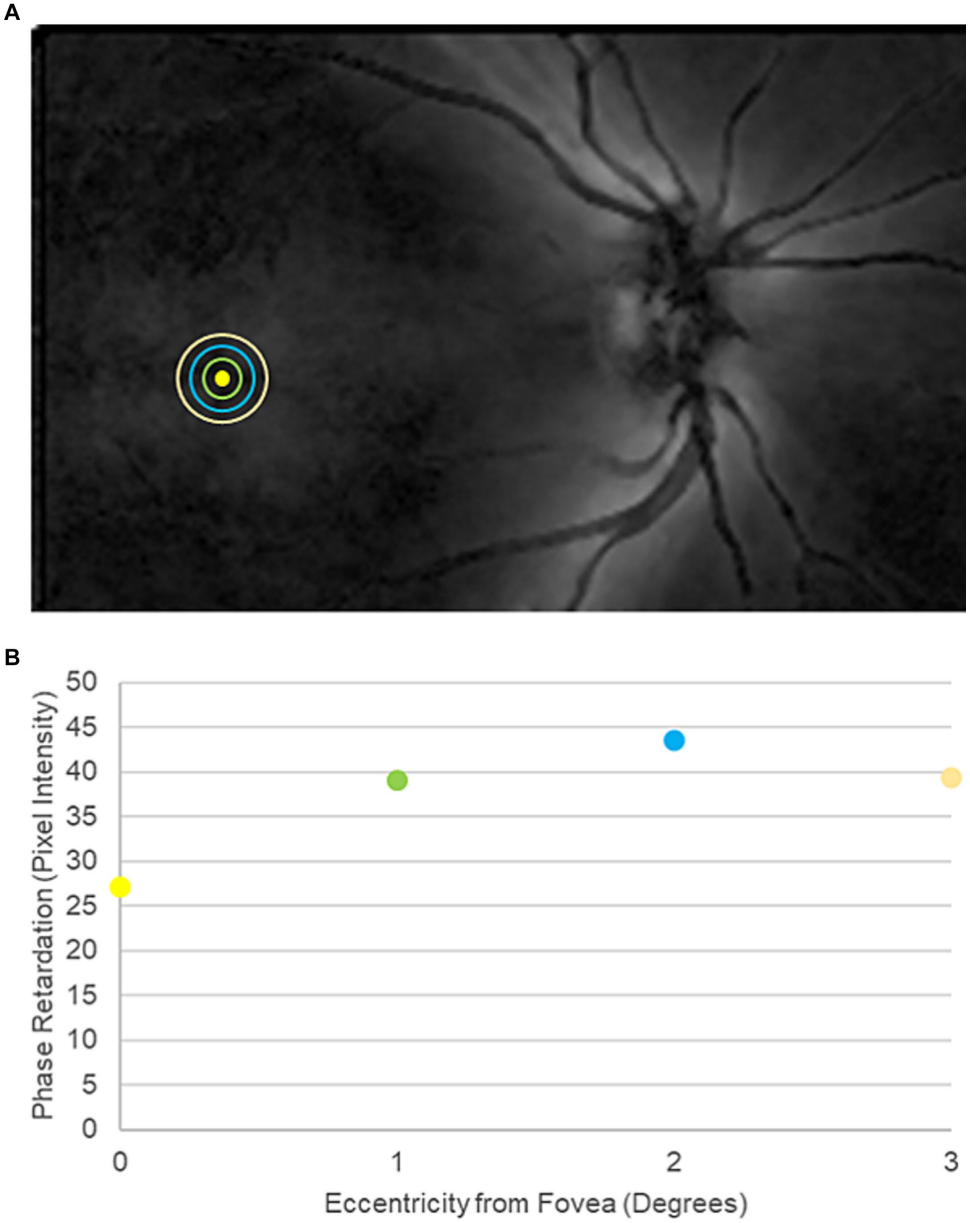
Representative scanning laser polarimetry (SLP) image from a participant in the traumatic brain injury cohort. **(A)** SLP image of the posterior pole. Bright regions within the image indicate high phase retardation caused by tissue birefringence. The yellow circle marks the center of the fovea. The green, blue, and orange rings indicate 1°, 2°, and 3° of eccentricity from the center of the fovea, respectively. **(B)** Macular phase retardation curve, comprising the fovea and the 1°, 2°, and 3° eccentric rings.

After OCT imaging, participants underwent SLP imaging with a single GDx device with variable corneal compensation (Laser Diagnostic Technologies, San Diego, CA) to measure phase retardation within the macula. Phase retardation is an optical signature of the HFL. A 20° × 40° macula-centered image was captured from each eye (Figure 2A).

##### 2.2.2.2 Image processing

Phase retardation maps with a quality score of ≥8 were exported from the device into MATLAB for pixel-intensity analysis. Specifically, pixel intensity, which indicates the magnitude of phase retardation at a given position, was measured globally across all points contained within the central 3° of the macula. Pixel intensity also was averaged at 1° concentric intervals from 1° to 3° eccentricity from the center of the fovea, matching the HFL thickness measurements from directional OCT (Figure 2B).

### 2.3 Electroretinography

Participants returned for dilated fERG at a second study session.

#### 2.3.1 Device and recording acquisition

Both eyes were anesthetized with proparacaine 0.5%, and Dawson, Trick, and Litzkow (DTL) Plus electrodes (Diagnosys; Lowell, MA) were placed in the lower conjunctival fornices. The DTL electrodes were referenced to skin electrodes placed near the ipsilateral temporal canthus of each eye, and a ground electrode was positioned at the center of the forehead. Participants then aligned with the Ganzfeld dome of a single Veris Pro (Electro-Diagnostic Imaging; Milpitas, CA) for fERG recordings in accordance with the International Society for Clinical Electrophysiology of Vision (ISCEV) (22), including, in order, the light-adapted (LA) 3.0, LA 30 Hz, dark-adapted (DA) 0.01, DA 3.0, and DA 10 conditions. Up to 20 repetitions were recorded under each condition with the goal of capturing 12 high-quality waveforms, save for 30 Hz flicker, which was recorded once. Inter-stimulus intervals were 1 s for the LA 3 condition, 2 s for the DA 0.01 condition, 10 s for the DA 3 condition, and 20 s for the DA 10 condition. The amplifier (15LT Physiodata Amplifier System with 15A54 Quad Amplifier; Grass Instrument Company; Quincy, MA) high- and low-cutoff frequencies were 1,000 Hz and 0.3 Hz, respectively.

#### 2.3.2 Waveform processing

**Figure 3:**
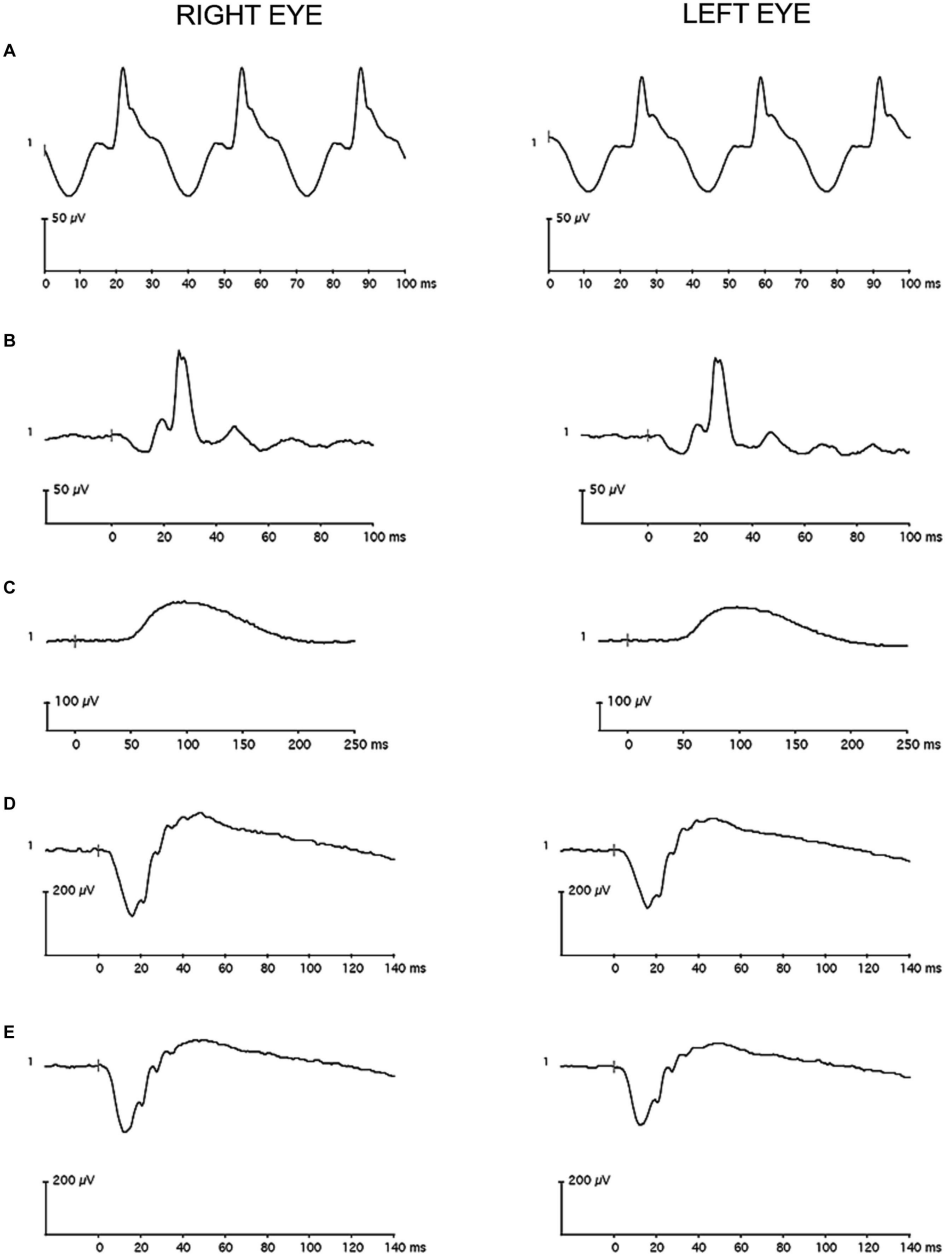
Representative full-field flash electroretinography recordings from a single control participant **(A)** for the light-adapted 30 Hz condition, **(B)** for the light-adapted 3.0 condition, **(C)** for the dark-adapted 0.01 condition, **(D)** for the dark-adapted 3.0 condition, and **(E)** for the dark-adapted 10 condition. The vertical gray line in the recordings marks flash onset.

**Figure 4:**
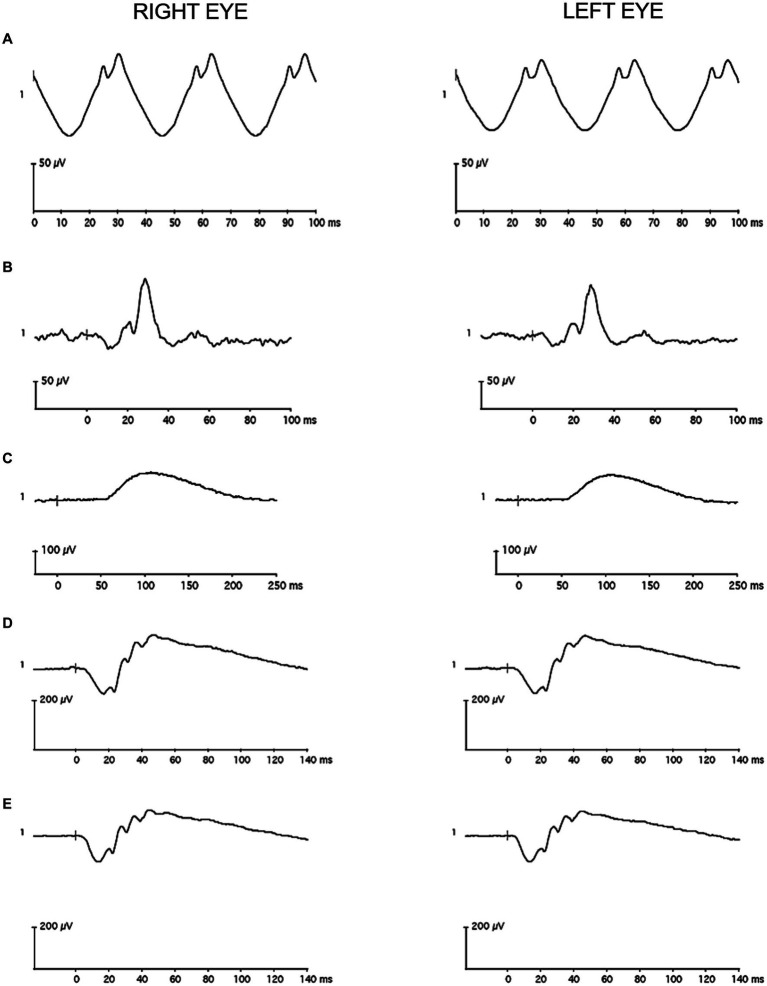
Representative full-field flash electroretinography recordings from a single case participant **(A)** for the light-adapted 30 Hz condition, **(B)** for the light-adapted 3.0 condition, **(C)** for the dark-adapted 0.01 condition, **(D)** for the dark-adapted 3.0 condition, and **(E)** for the dark-adapted 10 condition. The vertical gray line in the recordings marks flash onset.

A 60 Hz notch filter was not applied to the recordings, and drift removal was not utilized. Individual recordings were visually inspected, and those with artifacts were removed before analysis. The remaining waveforms were averaged in each eye. A single observer used commercial software on the device (version 6.4.5) to measure: a-wave amplitude and peak time for the LA 3.0, DA 3.0, and DA 10 conditions; b-wave amplitude and peak time for the LA 3.0, DA 0.01, DA 3.0, and DA 10 conditions; and amplitude and peak time for the LA 30 Hz condition. Figures 3, 4 contain representative waveforms from a single control participant and from a single case participant, respectively, for all test conditions.

### 2.4 Statistical analyses

#### 2.4.1 Retinal imaging

Consistent with a previous report in the literature (23), all imaging outcomes were averaged between the two eyes. If data from both eyes were unavailable, data from one eye were analyzed. Data from both eyes were collected from most participants, both for OCT (40 out of 50 participants) and for SLP (47 out of 49 participants).

Global HFL thickness was the primary outcome for directional OCT, and global phase retardation for SLP. HFL thickness and macular phase retardation at 1°, 2°, and 3° eccentricities and central foveal thickness were secondary outcomes for directional OCT and SLP, respectively. Imaging outcome measures were determined before data collection and were compared between the cohorts with two-tailed paired t-tests, if *p*-values from the Shapiro-Wilks test for normality were greater than 0.05. If Shapiro-Wilks *p*-values were less than 0.05, the data significantly deviated from a normal distribution, and Wilcoxon signed-rank test were used instead. The statistical significance threshold was preset at α = 0.05 for the primary outcomes. A Bonferroni correction was applied to the 45 secondary comparisons, including those from the imaging and ERG experiments, adjusting the statistical significance threshold for secondary outcomes to α = 0.0011.

The effect size (Cohen’s d) of each outcome was calculated as the mean of the inter-cohort difference divided by the standard deviation of the difference. In the absence of a good alternative, effect sizes for Wilcoxon signed-rank tests used the same calculation. When distribution is non-normal, Cohen’s d may be biased.

#### 2.4.2 Electroretinography

For each participant under each condition, amplitude values and peak time values were averaged between the eyes. If data from both eyes were unavailable, usually due either to blink artifacts or to technical difficulties, data from one eye were analyzed. Data from both eyes were collected from most participants for the LA 3.0 condition (42 out of 46 participants), for the LA 30 Hz condition (42 out of 42 participants), for the DA 0.01 condition (37 out of 44 participants), for the DA 3.0 condition (41 out of 44 participants), and for the DA 10 condition (40 out of 46 participants).

A-wave amplitude and peak time and b-wave amplitude and peak time of the LA 3.0 condition were the primary outcomes. These primary outcomes were determined before data collection and were selected for ease of future clinical implementation. All other fERG parameters were considered secondary outcomes. Two-tailed paired *t*-tests analyzed differences between the cohorts for normally distributed outcomes, and Wilcoxon signed-rank tests analyzed differences between the cohorts for non-normally distributed outcomes (α = 0.05 for the primary outcomes, and α = 0.0011 for the secondary outcomes). Cohen’s d was calculated for all outcome measures as a metric of effect size.

#### 2.4.3 Associations with TBI history

Associations between the primary imaging and fERG outcomes and TBI history, including total number of TBIs, years since last TBI, and years since first TBI, were quantified in the case cohort using Spearman’s Rank correlation coefficients tests (α = 0.0011).

#### 2.4.4 Structure–function relationships

Relationships between the primary imaging outcomes and the primary fERG outcomes in the case cohort were assessed with Pearson correlation coefficient tests (α = 0.0011).

## 3 Results

### 3.1 Study participants

Details about the study participants, including comprehensive TBIs histories, are reported in a companion study (16). Briefly, twenty-five (*n* = 25) case participants [mean ± standard deviation (SD) age = 32.2 ± 11.8 years; 52% female; 100% White] enrolled in the study. All case participants completed retinal imaging, and all but two returned for electroretinography. Case participants had a median (interquartile range) of 3 (3, 5) TBIs over a range of 0–41 years prior. Causes of TBI included falls, motor-vehicle accidents, strikes to the head, athletics, and assaults. Ninety-seven percent of the TBIs were classified as mild severity, and 3% were classified as moderate severity.

Thirty (*n* = 30) control participants (mean ± SD age = 34.4 ± 12.6 years; 47% female; 97% White and 3% Black) enrolled in the study. The OSU TBI-ID identified TBIs in four of the enrolled control participants, and optic nerve head drusen were discovered in another. These five participants (mean ± SD age = 44.0 ± 12.0 years; 20% female; 100% White) were excused before retinal imaging and electroretinography. The 25 remaining control participants, who completed retinal imaging after being pair-matched to case participants (Supplementary Table), had a mean ± SD age of 32.5 ± 12.0 years (52% female; 96% White and 4% Black). A single control participant (a 25–29 year-old white male) completed retinal imaging but did not return for electroretinography. Table 1 comprises medication lists for the case and control participants.

**Table 1:** Medications reported by study participants.

Medication class	Case cohort	Control cohort
Alpha-1 blocker	1	0
Analgesic	8	2
Angiotensin-converting enzyme inhibitor	1	0
Angiotensin receptor blocker	1	0
Antibiotic	0	2
Anticonvulsant	4	0
Antifungal	0	1
Antihistamine	2	4
Antimetabolite	1	0
Antiviral	0	1
Benzodiazepine	2	0
Beta-2 agonist	3	1
Beta blocker	1	0
Calcium channel blocker	1	0
Cholinesterase inhibitor	1	0
Leukotriene inhibitor	1	0
Hormonal	2	6
Multivitamin/mineral	7	6
Proton pump inhibitor	0	1
Selective serotonin reuptake inhibitor	5	2
Serotonin and norepinephrine reuptake inhibitor	4	0
Statin	1	0
Steroid	3	4
Stimulant	1	3
Succinate	1	0
Thiazide	1	0
Tricyclic/tetracyclic antidepressant	1	0

Values are numbers of medications reported by participants in the given cohort.

All study participants had monocular visual acuities of at least 20/25 using habitual refractive error correction. Refraction was not conducted as part of the study protocol, so best-corrected visual acuities were not measured. Case participants (mean ± SD spherical equivalent = −0.95 ± 1.62 D OD and − 0.90 ± 1.77 D OS) were significantly more myopic (*p* = 0.01, paired t-test) than control participants (−2.58 ± 1.90 D OD and − 2.74 ± 1.90 D OS), based on focimeter readings of habitual spectacles and on examination of previous medical records.

### 3.2 Retinal imaging

**Figure 5:**
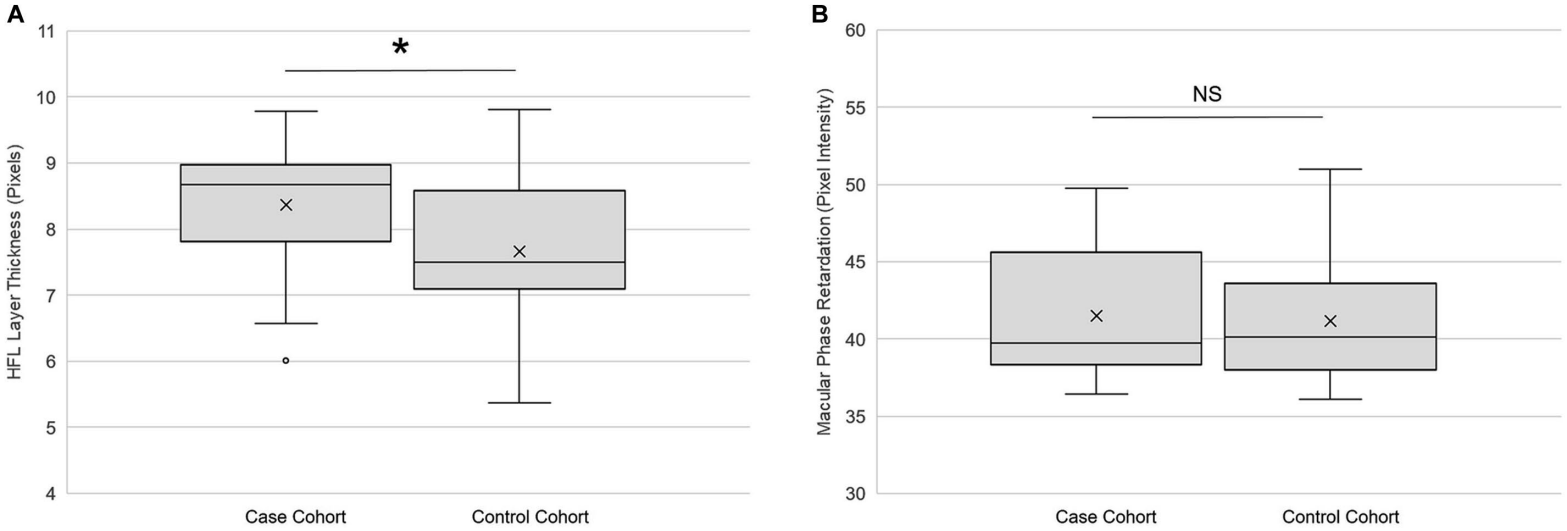
Retinal imaging primary outcomes. **(A)** Global Henle fiber layer thickness (*n* = 25) and **(B)** global macular phase retardation (*n* = 24) in both the case and the control cohorts. Each box represents the interquartile range, and the internal line is the median. The internal “X” is the mean. The whiskers represent the 90th and 10th percentiles, and open circles are outlying values. Phase retardation data for one case participant were not collected due to a technical difficulty. ^*^p < 0.05, paired t-test. NS is not statistically significant. Statistical significance threshold α = 0.05.

Global HFL thickness was significantly higher (*p* = 0.02, paired *t*-test) in cases than in controls (Figure 5A). The effect size (Cohen’s d) of 0.49 was medium. There was no statistically significant difference (*p* = 0.91, paired *t*-test) between the cohorts in global phase retardation in the macula (Figure 5B). The effect size of −0.02 was small. A qualitative review of the OCT and SLP images did not reveal areas of variable reflectance that might be indicative of an ischemic insult.

Table 2 contains the secondary outcomes of the retinal imaging studies. After Bonferroni correction, there were no statistically significant differences between the cohorts for HFL thickness at the eccentricities 1°, 2°, and 3° from the fovea; for macular phase retardation at the eccentricities 1°, 2°, and 3° from the fovea; and for central foveal thickness. However, the effect sizes of HFL thickness at the 2° and 3° eccentricities were of medium strength, as was the effect size for central fovea thickness.

**Table 2:** Secondary outcomes of the retinal imaging studies.

	TBI cohort	Control cohort	Paired mean difference	Effect size	*p*-value
**HFL thickness (pixels, *n* = 25)**					
1° eccentricity	8.0 ± 1.3	7.1 ± 1.6	0.8 ± 2.1	0.39	0.07
2° eccentricity	10.5 ± 1.5	9.3 ± 1.4	1.2 ± 1.9	0.62	0.01
3° eccentricity	9.9 ± 1.3	8.7 ± 1.6	1.2 ± 2.0	0.59	0.01^*^
**HFL phase retardation (pixel intensity, *n* = 24)**					
1° eccentricity	40.6 ± 4.1	40.4 ± 4.5	0.2 ± 5.6	0.03	0.88
2° eccentricity	47.8 ± 4.6	48.5 ± 4.6	−0.7 ± 6.7	−0.11	0.62
3° eccentricity	44.2 ± 4.6	45.2 ± 4.7	−0.9 ± 6.8	−0.14	0.52
Central foveal thickness (microns, *n* = 25)	230.5 ± 18.5	242.4 ± 20.7	−11.9 ± 22.3	−0.53	0.02

Henle fiber layer (HFL) phase retardation data from one case subject were lost due to technical difficulty. Values are mean ± standard deviation. Effect size is Cohen’s d. Comparisons were made with paired *t*-tests when the data were normally distributed. When they were not, the Wilcoxon signed-rank test was used instead (*). Statistical significance threshold α = 0.0011.

### 3.3 Electroretinography

**Figure 6:**
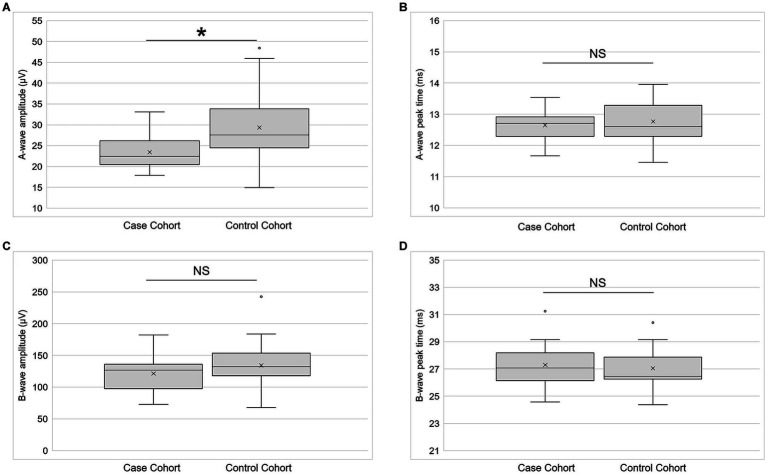
Electroretinography primary outcomes. **(A)** A-wave amplitude (*n* = 21), **(B)** a-wave peak time (*n* = 21), **(C)** b-wave amplitude (*n* = 21), and **(D)** b-wave peak time (*n* = 21) for both the case and control cohorts for the light-adapted 3.0 condition. Each box represents the interquartile range, and the internal line is the median. The internal “X” is the mean. The whiskers represent the 90th and 10th percentiles, and open circles are outlying values. Sample sizes vary because some participants did not return for the second study session or because their data did not meet quality control standards. ^*^p < 0.05, paired *t*-test. NS is not statistically significant. Statistical significance threshold α = 0.05.

LA 3.0 a-wave amplitude was significantly lower (*p* = 0.02; paired t-test) in cases than in controls (Figure 6A). The effect size (Cohen’s d) of −0.58 was medium. There was no statistically significant difference (*p* = 0.96; Wilcoxon signed-rank test) between the cohorts for LA 3.0 a-wave peak time (Figure 6B). The effect size was of −0.08 was small. Although LA 3.0 b-wave amplitude trended lower in cases than in controls (Cohen’s d = −0.22), the inter-cohort difference was not statistically significant (*p* = 0.32, paired *t*-test; Figure 6C). There also was no statistically significant difference between the cohorts for LA 3.0 b-wave peak time (*p* = 0.35, paired t-test; Figure 6D), but it trended longer in the case cohort than in the control cohort (Cohen’s d = 0.21).

Table 3 contains the secondary fERG outcomes. A-wave amplitude trended lower in cases than in controls under the DA 3.0 and DA 10 conditions, but the differences between the cohorts did not reach statistical significance. Likewise, b-wave amplitude for these conditions trended lower in cases than in controls, but the difference did not reach statistical significance. DA 0.01 peak time trended toward being shorter in cases than in controls, but the difference between cohorts was not statistically significant. There were no statistically significant differences between the cohorts for any of the other secondary fERG outcomes.

**Table 3:** Secondary outcomes of full-field flash electroretinography.

	TBI cohort	Control cohort	Paired mean difference	Effect size	*p*-value
**DA 0.01 condition**					
B-wave amplitude (μV, *n* = 19)	124.3 ± 40.9	148.3 ± 65.0	−24.0 ± 87.4	−0.27	0.25
B-wave peak time (ms, *n* = 19)	101.0 ± 5.8	104.8 ± 6.5	−3.8 ± 5.0	−0.75	0.004
**DA 3.0 condition**					
A-wave amplitude (μV, *n* = 19)	154.1 ± 31.6	193.7 ± 49.2	−39.6 ± 65.8	−0.60	0.06^*^
A-wave peak time (ms, *n* = 19)	15.7 ± 2.2	15.3 ± 0.5	0.4 ± 2.1	0.22	0.94^*^
B-wave amplitude (μV, *n* = 19)	283.2 ± 66.7	318.3 ± 88.8	−35.1 ± 118	−0.30	0.21
B-wave peak time (ms, *n* = 19)	48.2 ± 2.6	47.4 ± 2.6	0.8 ± 3.9	0.20	0.40
**DA 10 condition**					
A-wave amplitude (μV, *n* = 21)	182.2 ± 39.5	226.4 ± 58.2	−44.2 ± 76.1	−0.58	0.03^*^
A-wave peak time (ms, *n* = 21)	12.4 ± 1.3	12.5 ± 1.1	−0.2 ± 1.2	−0.15	0.49
B-wave amplitude (μV, *n* = 21)	294.8 ± 65.8	324.0 ± 88.9	−29.2 ± 117	−0.25	0.60^*^
B-wave peak time (ms, *n* = 21)	48.8 ± 2.9	47.8 ± 5.3	1.1 ± 6.1	0.18	0.69^*^
**LA 30 Hz condition**					
Amplitude (μV, *n* = 20)	92.0 ± 24.7	92.2 ± 26.9	−0.2 ± 33.8	−0.01	0.98
Peak time (ms, *n* = 20)	58.1 ± 1.8	57.6 ± 1.5	0.5 ± 1.7	0.29	0.21

DA is dark-adapted. LA is light-adapted. Sample sizes vary because some participants did not return for the second study session or because their data did not meet quality control standards. Values are mean ± standard deviation. Effect size is Cohen’s d. Comparisons were made with paired t-tests when the data were normally distributed. When they were not, the Wilcoxon signed-rank test was used instead (*). Statistical significance threshold α = 0.0011.

### 3.4 TBI associations

Table 4 contains associations between TBI history and the primary retinal imaging and fERG outcomes in the case cohort. Most of the associations were modest in magnitude, and none reached statistical significance. There were medium-strength negative associations between number of lifetime TBIs and LA 3.0 a-wave peak time and between number of lifetime TBIs and LA 3.0 b-wave peak time. Neither of these medium-strength associations reached statistical significance. All other associations between TBI and the primary retinal imaging and fERG outcomes in the case cohort were weak and not-statistically-significant.

**Table 4:** Associations between traumatic brain injury (TBI) history and retinal structure and function.

	Spearman’s rank correlation coefficient	*p*-value
**Number of TBI, vs.**		
LA 3.0 a-wave amplitude (μV, *n* = 22)	0.06	0.78
LA 3.0 b-wave peak time (ms, *n* = 22)	−0.46	0.03
LA 3.0 b-wave amplitude (μV, *n* = 22)	0.08	0.74
LA 3.0 b-wave peak time (ms, *n* = 22)	−0.38	0.08
Global HFL thickness (pixels, *n* = 25)	0.09	0.67
Global HFL phase retardation (pixel intensity, *n* = 24)	0.20	0.36
**Years since last TBI, vs.**		
LA 3.0 a-wave amplitude (μV, *n* = 22)	0.24	0.28
LA 3.0 a-wave peak time (ms, *n* = 22)	0.29	0.19
LA 3.0 b-wave amplitude (μV, *n* = 22)	0.04	0.88
LA 3.0 Bb-wave peak time (ms, *n* = 22)	0.27	0.23
Global HFL thickness (pixels, *n* = 25)	0.03	0.89
Global HFL phase retardation (pixel intensity, *n* = 24)	−0.09	0.68
**Years since first TBI, vs.**		
LA 3.0 a-wave amplitude (μV, *n* = 22)	0.06	0.80
LA 3.0 a-wave peak time (ms, *n* = 22)	−0.05	0.84
LA 3.0 b-wave amplitude (μV, *n* = 22)	−0.25	0.25
LA 3.0 b-wave peak time (ms, *n* = 22)	0.09	0.69
Global HFL thickness (pixels, *n* = 25)	0.15	0.48
Global HFL phase retardation (pixel intensity, *n* = 24)	0.00	0.99

LA is light-adapted. HFL is Henle fiber layer. Sample sizes vary because some participants did not return for the second study session or because their data did not meet quality control standards. Statistical significance threshold α = 0.0011.

### 3.5 Structure–function associations

In the case cohort, there were no statistically significant relationships between global HFL thickness and LA 3.0 a-wave amplitude (r = 0.06, *p* = 0.72, Pearson correlation), LA 3.0 a-wave peak time (r = −0.07, *p* = 0.65), LA 3.0 b-wave amplitude (r = 0.05, *p* = 0.72), or LA 3.0 b-wave peak time (r = 0.15, *p* = 0.34). Likewise, there were no statistically significant relationships between global HFL phase retardation and LA 3.0 a-wave amplitude (r = −0.17, *p* = 0.25, Pearson correlation), LA 3.0 a-wave peak time (r = −0.06, *p* = 0.67), LA 3.0 b-wave amplitude (r = −0.07, *p* = 0.67), or LA 3.0 b-wave peak time (r = −0.15, *p* = 0.33).

## 4 Discussion

The purpose of this study was to test the hypotheses that multiple TBIs alter the structure of the HFL and that they impair the objective function of the retina in human participants.

### 4.1 Henle fiber layer thickness

Directional OCT measured the thickness of the HFL, and SLP assessed the structural integrity of its photoreceptor axons. Global HFL thickness was significantly greater in cases cohort, compared to controls. This thickening became pronounced outside of the central fovea, with the largest effect sizes occurring at 2° and 3° eccentricities. There was a smaller effect at 1° eccentricity, where the HFL thickness is small enough for normal variation among individuals to obscure differences between groups. There was no statistically significant difference in central foveal thickness between the cohorts, but there was a trend toward thinning in the case participants (Cohen’s d = −0.53). Future studies should be designed to assess the relationships between the HFL thickness and other measures of macular structure.

HFL thickening after TBI is a novel finding but aligns with a study that described non-layer-specific macular swelling in boxers after 18 months of training (8). Beyond the macula, Gilmore and colleagues found baseline RNFL thickening in veterans with a history of TBI, compared to non-concussed veterans (23). We previously reported that there were no differences in RNFL thickness between the cohorts in the present study (16), suggesting that the macula and the HFL may be better sites to detect post-TBI retinal changes than the RNFL, perhaps due to fewer large blood vessels and astrocytes in the former vs. the latter (24). Evidence in support of this possibility can be found in early-stage glaucoma, where macular thinning may be detectable before RNFL thinning (25, 26).

The etiology of increased HFL thickness in cases compared to controls is unclear. Case series have reported increased tortuosity of retinal blood vessels and the presence of hemorrhages in the HFL in patients with acute TBI (27, 28). The HFL contains portions of the deep capillary plexus (DCP) (29), a major conduit for venous outflow in the retina (30). It is possible that similar changes to the structure of and to the permeability of the DCP chronically accumulate in patients with multiple TBIs, causing the HFL to swell. OCT angiography technology will enhance the ability of future studies to quantify macular profusion after TBI. Alternatively or additionally, the HFL contains the processes of Müller cells (31). Müller cells change shape and increase their volume in response to acute and chronic insults (31, 32). HFL thickening in aging maculae, similar to that described here, has been attributed to this process (33), raising the possibility that multiple TBIs elicit retinal pathology akin to early aging. Our study population was young, on average, and we cannot extrapolate our results to older populations at this time. It will be important for future studies to establish the specificity of HFL thickening as a potential maker of TBI pathology by differentiating the attributes of HFL thickening after TBI from those of HFL thickening due to aging.

### 4.2 Henle fiber layer phase retardation

In contrast to HFL thickness, there was no statistically significant difference in global phase retardation between the two cohorts. Nor were there any statistically significant differences at eccentricities 1°, 2°, or 3° from the central fovea. This finding suggests that the birefringent microtubules of the photoreceptor axons remained intact within the thickened HFL in the case cohort. It also coincides with our previous report that the phase retardation of the RNFL did not differ between the two cohorts (16).

### 4.3 Electroretinography

We used fERG to objectively test whether function was altered in the remaining photoreceptors and their bipolar cells. The a-wave amplitude of the LA 3.0 fERG was significantly reduced in cases compared to controls. Furthermore the a-wave amplitude under DA 3.0 (Cohen’s d = −0.60) and DA 10.0 (Cohen’s d = −0.58) conditions trended lower in the cases than in the controls. The DA 3.0 a-wave reflects the activity of rods, and the LA 3.0 a-wave mainly reflects the OFF-cone bipolar cell responses, with direct contributions from cone currents at higher flash strengths. A reduction in a-wave amplitude across multiple testing conditions therefore signals an impairment of photoreceptor function after multiple TBIs. Future investigations will need to determine the cause of this impairment, but the same factors that may elicit HFL thickening also may undermine the function of photoreceptors.

Even though b-wave amplitude consistently trended lower in cases than in controls, the inter-cohort difference never reached statistical significance under any testing condition. These results suggest little difference in ON bipolar cell function between the cohorts, and they were surprising, given the reduction in a-wave amplitude. Several factors may have contributed. The case participants were likely more photosensitive than their pair-matched controls (34), which made it challenging to record blink free responses under some testing conditions. This variable may have especially influenced the DA 0.01 b-wave peak time, which occurs after the latency period for blink initiation after a light stimulus (35). It may have also affected the outcomes of the visually demanding LA 30 Hz condition, a summation of ON- and OFF-cone bipolar cell responses that reflects cone pathway function.

Our choice of fERG stimuli also may have influenced the b-wave results. We did not detect statistically significant differences between the cohorts in b-wave amplitude using the relatively bright settings of the ISCEV standard protocol for fERG. Future studies should consider using the ISCEV extended protocol for the stimulus–response series for the dark-adapted fERG b-wave to detect differences in bipolar cell function between TBI and control participants (36). This protocol employs a series of increasing stimulus strengths to provide a measure of retinal sensitivity, according to a heuristic model. Al-Abdalla and colleagues used a modified version of it to show that the slope and the semi-saturation constant of the Naka-Rushton equation describing the photopic negative response are altered in participants with mild TBI, compared to controls, even though the photopic negative response amplitude did not differ between the groups (37). The authors did not find differences between the cohorts in b-wave characteristics, but this finding may be due to the unique red-on-blue stimuli used to drive cone responses to maximize the photopic negative response.

### 4.4 Clinical considerations

The current study was designed to facilitate future clinical applications. The retinal imaging instruments that were used are all commercially available, and the method that was used to determine location within the macula (i.e., using angular distances and not linear distances) is consistent with the data analysis software packages on these devices. Although the ERG system that was used is geared toward research, the primary outcome measures for the functional experiments (i.e., those generated during LA 3.0 condition) would likely be the easiest to run in the clinic on other systems.

Despite these steps to promote the translational nature of our results, there is more work to be done before clinical implementation. First, the data collection techniques that we used are technically challenging. Directional OCT requires dilated pupils, steady fixation, and well-trained technicians. Additionally, not all OCT imaging devices have the capacity to move their imaging beams in such a manner as to acquire the off-axis images necessary to visualize the HFL; and even if they did, there are currently no normalized HFL datasets against which to compare individual results. ERG systems face many of the same challenges as directional OCT, but new clinically-oriented devices are now facilitating easy and efficient data collection in the clinic.

Beyond these technical issues, more needs to be learned about the structure and function of the retina after TBI before it can be used as a site to reliably and objectively detect pathology. Specifically, more longitudinal studies are needed to document how retinal structure and function change over time after a TBI. Our cross-sectional study was performed on participants with chronic TBI; it is unknown whether the results would be similar in the acute stage of the condition. It will also be necessary to determine how specific they are to TBI, compared to normal aging and to other neurodegenerations.

### 4.5 Study limitations

There are several limitations to this study. First, even though our study population comprised males, females, and case participants with diverse TBI histories, it was small. Second, macular thickness and the ERG waveform vary by race (38, 39); therefore, the generalizability of our results may be limited by the fact that our study population was overwhelmingly White. The facts that case participants fit a specific injury profile (i.e., multiple mid-moderate TBIs over a lifetime) and that most case participants were experiencing visual symptoms after their TBIs may also limit the generalizability of our results. Future research should try to replicate our results in diverse populations, including those with high incidences of TBI and individuals with suspected chronic traumatic encephalopathy. Third, our OCT instrument was unable to measure macular ganglion cell layer thickness; therefore, we were unable to test whether multiple TBIs alter the structure of this part of the retina. As assessment of the ganglion cell layer is a critical component of the retinal assessment of other neurodegenerations, such as glaucoma (40) and Alzheimer’s disease (41), it should be included in future studies of retinal structure after TBI. Fourth, fERG measures gross retinal function, limiting its ability to test structure–function relationships within the HFL. It is therefore unsurprising that we did not detect significant relationships between the HFL and fERG outcomes. Future studies could use multi-focal ERG to target function in the central macula for comparison against the structure of the HFL. Fifth, we did not thoroughly assess visual function beyond perimetry and beyond habitual visual acuity. Future studies are necessary to test the hypothesis that the objectively measured changes in retinal structure and function in individuals with a history of multiple TBIs are associated with deficits in best-corrected visual acuity, contrast sensitivity, color vision, and other visual performance parameters.

Finally, although we did not measure axial length as part of the study protocol, the case cohort was more myopic than the control cohort by approximately 1.5 D, as previously reported (16), meaning that axial length was likely longer in the former than in the latter. We did not account for this probable difference in axial length during retinal-image processing, which means that the linear distances between the 1°, 2°, and 3° eccentric measurements of HFL thickness and of macular phase retardation differed between the cohorts. The effect that these linear differences had on our data was likely small; for Chui and colleagues reported that photoreceptor density, which we assume is proportional to the HFL thickness, is uniform across different axial lengths when measured at angular distances (42). Thus, the use of angular distances in this study allowed inter-group comparisons to be straightforward and clinically applicable without incurring a negative effect. To confirm, linear regression analysis was used to test for associations between refractive error, as a proxy for axial length, and HFL thickness and between refractive error and HFL phase retardation. For the combined data of the case and control cohorts, there were no significant associations between spherical equivalent refractive error and global HFL thickness (r = 0.04, *p* = 0.78) and between spherical equivalent refractive error and global HFL phase retardation (r = 0.06, *p* = 0.72). These results suggest that the difference in refractive error between the cohorts did not impact our imaging outcomes.

## 5 Conclusion

In conclusion, participants with a history of multiple TBIs had a thicker HFL than controls. The former also had reduced a-wave amplitudes during the LA 3.0 fERG, suggesting a deficit in photoreceptor function. Currently, there are no objective methods to diagnose TBI. Our results support the possibility that the retina is a site to detect and follow the pathology associated with TBI using equipment available in eye clinics around the world.

## Data availability statement

The raw data supporting the conclusions of this article will be made available by the authors, without undue reservation.

## Ethics statement

The studies involving humans were approved by the Ohio State University Biomedical Institutional Review Board. The studies were conducted in accordance with the local legislation and institutional requirements. The participants provided their written informed consent to participate in this study.

## Author contributions

ES-G: Data curation, Writing – review & editing. KK: Data curation, Writing – review & editing. ED: Data curation, Writing – review & editing, Investigation. ES: Data curation, Writing – review & editing, Investigation. MR: Formal analysis, Writing – review & editing. LJ: Formal analysis, Methodology, Writing – review & editing. JR: Formal analysis, Methodology, Writing – review & editing. DV: Data curation, Formal analysis, Funding acquisition, Resources, Writing – review & editing, Software. CM: Investigation, Methodology, Writing – review & editing. PY: Conceptualization, Data curation, Formal analysis, Funding acquisition, Investigation, Methodology, Project administration, Supervision, Writing – original draft, Writing – review & editing.
