# Supplementary material for: Henle fiber layer thickening and deficits in objective retinal function in participants with a history of multiple traumatic brain injuries
Source: Front Neurol. 2024 Feb 6;15:1330440. doi: 10.3389/fneur.2024.1330440 (PMC10876769; doi:10.3389/fneur.2024.1330440)
Supplement: Supplementary file 1 [file Table_1.DOCX]

Supplementary Material

Henle Fiber Layer Thickening and Deficits in Objective Retinal Function in Participants with a History of Multiple Traumatic Brain Injuries

**Elizabeth A. Stern-Green, Kelly R. Klimo, Elizabeth Day, Erica R. Shelton, Matthew L. Robich, Lisa A. Jordan, Julie Racine, Dean A. VanNasdale, Catherine E. McDaniel, Phillip T. Yuhas***

*** Correspondence:** Phillip T. Yuhas: Yuhas.10@osu.edu

Supplemental Table. Pair-matched study participants.

| Cases |  |  | Matched Controls |  |  |
| --- | --- | --- | --- | --- | --- |
| Participant number | Age range (years) | Sex | Participant number | Age range (years) | Sex |
| 101 | 50-54 | F | 226 | 50-54 | F |
| 102 | 20-24 | M | 204 | 25-29 | M |
| 103 | 30-34 | M | 218 | 30-34 | M |
| 104 | 25-29 | F | 209 | 30-34 | F |
| 105 | 20-24 | F | 202 | 20-24 | F |
| 106 | 20-24 | M | 212 | 25-29 | M |
| 107 | 25-29 | F | 201 | 25-29 | F |
| 108 | 25-29 | F | 208 | 25-29 | F |
| 109 | 25-29 | F | 205 | 20-24 | F |
| 110 | 25-29 | F | 210 | 25-29 | F |
| 111 | 25-29 | F | 206 | 20-24 | F |
| 112 | 20-24 | F | 215 | 25-29 | F |
| 113 | 50-54 | M | 221 | 50-54 | M |
| 114 | 15-19 | M | 220 | 20-24 | M |
| 115 | 25-29 | F | 207 | 20-24 | F |
| 116 | 55-59 | F | 227 | 55-59 | F |
| 117 | 40-44 | M | 228 | 40-44 | M |
| 118 | 50-54 | M | 230 | 50-54 | M |
| 119 | 40-44 | M | 229 | 40-44 | M |
| 120 | 25-29 | M | 203 | 25-29 | M |
| 121 | 20-24 | M | 213 | 25-29 | M |
| 122 | 50-54 | M | 223 | 50-54 | M |
| 123 | 25-29 | F | 211 | 25-29 | F |
| 124 | 15-19 | M | 214 | 20-24 | M |
| 125 | 25-29 | F | 216 | 25-29 | F |

F is female. M is male. Control participants 217, 219, 222, 224, and 225 were dismissed from the study before data collection and thus were not paired with case participants.
